# Biofeedback interventions for people with cerebral palsy: a systematic review protocol

**DOI:** 10.1186/s13643-017-0405-y

**Published:** 2017-01-13

**Authors:** Alexander MacIntosh, Nicolas Vignais, Elaine Biddiss

**Affiliations:** 1 Institute of Biomaterials and Biomedical Engineering, University of Toronto, Toronto, ON Canada; 2 Bloorview Research Institute, Holland Bloorview Kids Rehabilitation Hospital, 150 Kilgour Road, Toronto, ON M4G 1R8 Canada; 3 CIAMS, Univ. Paris-Sud, Université Paris-Saclay, 91405 Saint-Aubin, Orsay Cedex France; 4 CIAMS, Université d’Orléans, 45067 Orléans, France

**Keywords:** Sensory feedback, Visual feedback, Audio feedback, Haptic feedback, Neurofeedback, Cues, Hemiplegia, Spastic diplegia, Motor rehabilitation

## Abstract

**Background:**

Cerebral palsy is a life-long disability that affects motor control and activities of daily living. Depending on the type of cerebral palsy, some individuals may have trouble performing tasks with one or both of their arms and/or legs. Different strategies exist to help develop motor capacity. Biofeedback therapy is a commonly applied rehabilitation strategy. In biofeedback therapy, information about the motor behavior while completing a task is given back to the individual to help improve their performance. This can provide valuable information that would otherwise be unknown to the individual. Biofeedback may also have a unique method of operation in clinical populations, such as people with cerebral palsy. Therefore, it is important to identify the most effective mechanisms for specific populations. This review aims to evaluate the effects of biofeedback interventions that have been used towards improving motor performance and motor learning in people with cerebral palsy.

**Methods:**

Using a customized strategy, MEDLINE, CINAHL, Embase, PsycINFO, Cochrane Central Register of Controlled Trials, SCOPUS, SPORTDiscus, and PEDro databases will be searched. Two independent reviewers will screen titles and abstracts, review full texts for inclusion criteria, and extract data from relevant articles using a standardized template. Quality of evidence and risk of bias will be assessed through the Grading of Recommendations Assessment, Development, and Evaluation (GRADE) methodology.

**Discussion:**

Several studies have investigated biofeedback-based interventions for people with cerebral palsy. However, there is a great variety and limited consensus regarding how to implement biofeedback mechanisms. This systematic review will consolidate the current evidence to direct future study and develop effective biofeedback rehabilitation strategies.

**Systematic review registration:**

PROSPERO ID: CRD42016047612

**Electronic supplementary material:**

The online version of this article (doi:10.1186/s13643-017-0405-y) contains supplementary material, which is available to authorized users.

## Background

### Description of condition

Cerebral palsy (CP) is a common disability related to an injury or abnormality of the brain occurring near birth which persists from childhood through adulthood [1]. CP can impact a person’s motor control, perception, intellectual function, ability to perform daily activities (e.g., walking, eating), and participation in society [2]. The condition is generally classified based on the affected body region(s) (e.g., hemiplegia, diplegia) and the type of tone or movement abnormality (e.g., spasticity, dyskinesia). However, there is a great overlap and variability across the spectrum of individuals with CP [1]. Based on the different CP subtypes, individuals may have impaired motor control in one or both of their arms and/or legs.

Intervention strategies for improving motor function often involve intensely using of the non-dominant limb. For example, during constraint-induced movement therapy (CIMT), the dominant hand is cast or covered to force the use of the non-dominant hand for all activities [3]. Similarly, bimanual training requires greater than normal use of the non-dominant hand by asking the individual to complete more tasks that require both hands [4]. These types of intervention strategies are based in principles of motor learning, where the motor patterns reinforced during therapy aim to influence the person’s ability to reproduce a skill independently [5]. Motor learning is thought to occur as a result of an adapted central nervous system, or neuroplastic change as a result of the rehabilitation experiences that demand a high frequency and intensity of the practice [6]. The intervention effectiveness can be modulated by a number of factors including task specificity, timing, and feedback [6, 7]. Feedback of a movement is also critical when implementing strategies to improve motor performance and motor learning because of its influence on recall and recognition in memory [7, 8]. Given the complexity of cerebral palsy, it is essential to understand how feedback can be effectively utilized to instill changes in motor function.

### Description of intervention

Feedback in motor performance can be “task-intrinsic” (or inherent), that is from the natural perception within the individual. Alternatively, feedback can be from an external source. External feedback may be information from a therapist or device and can be given to the individual during or at the end of the task [7]. In this review, biofeedback refers to external feedback, where information about the motor performance is communicated back to the individual.

Biofeedback can be used to represent any biological variable, for example, the arm orientation while reaching [9], muscle activity patterns during walking [10], or changes in center of mass while running [11]. This information can be delivered in a variety of ways such as visual, audio, and haptic cues [12]. In a clinical population, biofeedback may operate differently than in a typical population [9]. In addition to facilitating motor learning by engaging auxiliary sensor inputs, biofeedback may enhance compensatory strategies required to overcome loss of function [9]. It is particularly important in CP, where the extent of the injury can vary so widely, to identify biofeedback interventions effective in improving motor performance and motor learning.

### Purpose of review

Several studies have investigated motor therapies that employ biofeedback, e.g. [10–14]. However, the diversity of task objectives and biofeedback parameters make conclusions regarding the effects of biofeedback for people with CP difficult to synthesize. Therefore, this review aims to evaluate the effects of biofeedback interventions used towards improving motor performance and motor learning in people with CP.

### Methods/design

This protocol was developed in accordance with the Preferred Reporting Items for Systematic Reviews and Meta-Analyses Protocols (PRISMA-P). The PRISMA-P checklist has been included in an additional file (see Additional file 1) [15]. The systematic review has been registered with PROSPERO (Ref: CRD42016047612).

### Study selection criteria

Any quantitatively study design, published in English or French, evaluating a biofeedback intervention, e.g.:

1. Randomized controlled trials (RCTs)
2. Quasi-randomized trials
3. Quasi-experimental studies
4. Cross-over trials
5. Case-control studies
6. Cohort studies
7. Case series (*N* > 3)
8. Published conference papers

### Participant inclusion

1. Clinical diagnosis of CP.
2. There is no restriction on the severity or stage of the disease.
3. Exclusion: comorbidities identified by the authors as potentially obscuring results of the biofeedback intervention (e.g., severe cognitive impairment).

### Interventions

1. Therapeutic intervention applied to improve the following:
  - Motor function (control, strength, daily activities)
  - Indications (e.g., spasticity, dystonia, hypertonicity)
  - Kinematics (e.g., speed, body trajectory)
  - Kinetics (e.g., applied force)
2. Using external feedback related to the participant’s movement, e.g.:
  - Electromyography (EMG) (e.g., co-contraction, synergy patterns)
  - Kinematic (e.g., speed, body trajectory)
  - Kinetic (e.g. applied force)
3. Returning feedback to the participant using through external stimuli, e.g.:
  - Visual
  - Auditory
  - Haptic

### Comparators

1. No treatment/control group
2. “Sham”/active control
3. Pre-post/self-control

### Outcomes

1. The primary outcomes will relate to motor performance and/or motor learning, with implications for improving daily activity, clinical measures, and functional tasks, e.g.:
  - Quality of upper extremity skills test (QUEST) [16]
  - Jebsen–Taylor hand function test [17]
  - Gross motor function classification system (GMFCS) [18]
  - Manual ability classification system (MACS) [19]
  - Described functional tasks
2. Secondary outcomes will relate to qualitative and quantitative measures of task performance, e.g.:
  - Kinematics (movement speed, trajectory, range of motion, error)
  - Kinetics (center of pressure, strength)
  - EMG (co-contraction, normalized muscle activity)
  - Participant/guardian-determined evaluations of efficacy

### Search Strategy

Relevant articles will be identified through the following databases: MEDLINE, CINAHL, Embase, PsycINFO, Cochrane Central Register of Controlled Trials, SCOPUS, SPORTDiscus, PEDro. References of relevant articles will also be searched for additional studies. An example of the search strategy can be found in the Appendix.

## Data collection and analysis

### Study selection

One reviewer will complete all database searches. Two reviewers will independently screen titles and abstracts to identify potentially relevant article and then review full texts of the relevant articles to determine eligibility. Any disagreements will be resolved through discussion, and if necessary, a third reviewer will be consulted.

### Data extraction and management

Data will be managed in DistillerSR systematic review software (https://www.evidencepartners.com). Data from eligible studies will be extracted by two reviewers independently using a customized checklist. Extracted data items will include the following:

1. Study design
  - Study type (e.g., RCT, crossover, case series)
  - Randomization
  - Blinding
  - Inclusion/exclusion criteria
2. Participant characteristics of each group
  - Number of participants
  - CP sub-type/description
  - Comorbidities
  - Gender
  - Age (median, mean)
3. Intervention characteristics
  - Biofeedback description
    i. Variables used to create feedback
    ii. Methods of modulation
    iii. Method of presentation
      1. Modality (e.g., visual, audio, haptic)
      2. Timing (concurrent vs terminal)
  - Duration and frequency of intervention
4. Analysis
  - Outcome measures
    i. Timing of evaluation (e.g., pre-post)
  - Statistical tests performed
  - Comparative group (pre-post, control)
5. Results of relevant outcomes
  - Mean group/period scores
  - Statistical/clinical significance identified by the authors
6. Adverse effects
7. Author conflict of interest statement

### Risk of bias

The Grading of Recommendations Assessment, Development, and Evaluation (GRADE) will be used by two independent reviewers to assess risk of bias [20]. Risk of bias will be assessed using key criteria identified by the GRADE approach. For randomized studies, key criteria include lack of allocation/concealment, lack of blinding, incomplete accounting of outcome or patient events, and selective outcome reporting. For non-randomized studies, key criteria include inappropriate eligibility, flawed measurements of exposure and outcome, not controlling for confounding factors, and inadequate follow-up (Tables 5.4 and 5.5 of the GRADE Handbook) [21]. Additionally, where possible, individual RCTs will be assessed using the Cochrane Risk of Bias Tool [22]. These individual ratings will be used through the GRADE methodology to evaluate the study limitations when determining the quality of evidence for each outcome measure. Disagreements will be resolved through discussion, and if necessary, a third reviewer. Any concerns of within-study or publication bias will be reported.

### Quality of evidence

The level of evidence for primary and secondary outcome measures will be evaluated using the GRADE working group methodology. Outcomes will be given one of four levels from high to very low based on five criteria: study design, risk of bias, consistency, directness, and precision as per GRADE methodology [20].

### Data synthesis

Outcome measures from each study will be categorized as either primary, that is related to functional performance metrics of daily activities, or categorized as secondary, relating to physiological changes during controlled tasks (e.g., improved movement speed or accuracy). Within the primary and secondary outcome categories, individual outcomes will be sub-grouped to the extent possible based on their potential patient impact. A summary of findings with the related quality of evidence for each of these sub-grouped outcomes will be synthesized to reflect the current state of biofeedback interventions aiming to improve motor performance for people with cerebral palsy.

## Discussion

Improved motor performance and autonomy during activities of daily living is a focus of rehabilitation interventions for people with CP. Biofeedback plays an integral role in the development of motor performance and motor learning and can be incorporated into many rehabilitation strategies. As such, the proposed systematic review will evaluate the efficacy of biofeedback interventions to direct future study and rehabilitation strategies. This review can also be considered as a guide for determining the impact of biofeedback interventions.

## Appendix

Sample search terms used in EMBASE

1. sensory feedback/or auditory feedback/or proprioceptive feedback/or tactile feedback/or visual feedback/or neurofeedback/or constructive feedback/or feedback system/or association/or reinforcement/
2. (((sensor* or audit* or visual* or tactile* or propriocept* or haptic*) adj3 (cue* or reinforce* or feedback*)) or “biofeedback*” or neurofeedback* or (feedback* adj3 (construc* or system*))).tw.
3. 1 or 2
4. cerebral palsy/or hemiplegia/or quadriplegia/or ataxic gait/or athetosis/or dystonia/or dyskinesia/or choreoathetosis/or spasticity/
5. (cereb* adj3 pals*) or monopleg* or dipleg* or tripleg* or quadripleg* or hemipleg* or spastic* or dystoni* or ataxi* or atheto* or dyskine* or chorea*.tw.
6. 4 or 5
7. 3 and 6

## Additional file

Additional file 1: Microsoft word document. PRISMA-P 2015 Checklist. This additional file is the completed PRISMA-P checklist used for the development of the protocol. (DOCX 31.7 kb)

## Abbreviations

CP: Cerebral palsy
CIMT: Constraint-induced movement therapy
EMG: Electromyography
GMFCS: Gross motor function classification system
GRADE: Grading of Recommendations Assessment, Development, and Evaluation
MACS: Manual ability classification system
PRISMA-P: Preferred Reporting Items for Systematic Reviews and Meta-Analyses Protocols
QUEST: Quality of upper extremity skills test
RCTs: Randomized controlled trials
